# Challenges in the treatment of *BRAF* K601E-mutated lung carcinoma: a case report of rapid response and resistance to dabrafenib and trametinib

**DOI:** 10.3389/fonc.2024.1374594

**Published:** 2024-07-08

**Authors:** Akihiro Nishiyama, Shigeki Sato, Hiroyuki Sakaguchi, Hiroshi Kotani, Kaname Yamashita, Koushiro Ohtsubo, Shigeki Nanjo, Seiji Yano, Keishi Mizuguchi, Hiroko Ikeda, Shinji Takeuchi

**Affiliations:** ^1^ Department of Medical Oncology, Kanazawa University Hospital, Kanazawa, Japan; ^2^ Department of Respiratory Medicine, Kanazawa University Hospital, Kanazawa, Japan; ^3^ Department of Diagnostic Pathology, Kanazawa University Hospital, Kanazawa, Japan

**Keywords:** BRAF K601E mutation, lung carcinoma, dabrafenib, trametinib, resistance to targeted therapy, liquid biopsy

## Abstract

We report a case of limited effectiveness of dabrafenib and trametinib in a 59-year-old man with poorly differentiated lung carcinoma and a rare *BRAF* K601E mutation. The patient, unresponsive to chemotherapy and immunotherapy, received these targeted agents as second-line treatment. Despite a notable initial response, tumor regression lasted only 52 days. A subsequent liquid biopsy revealed additional alterations (*BRAF* amplification, *KIT* amplification, *TP53* S241F), indicating a complex resistance mechanism. This case underscores the challenges in treating *BRAF* K601E-mutant lung carcinoma, emphasizing the need for advanced molecular diagnostics, personalized approaches, and further research into more effective therapies for unique genetic profiles.

## 1 Introduction

*BRAF* mutations are recognized as tumor-agnostic driver mutations that play pivotal roles in the pathogenesis of various cancers (1). Among the more than 50 identified *BRAF* mutations, the V600E variant is the most prevalent, accounting for approximately 63% of all *BRAF* mutations (1). In non-small cell lung cancer (NSCLC), these mutations occur in approximately 3% of cases, with one-third involving the V600E mutations. Targeted therapy, utilizing a BRAF inhibitor such as dabrafenib in combination with a MEK inhibitor like trametinib, is approved for melanoma and NSCLCs with the *BRAF* V600E mutation. However, the treatment landscape for non-V600E mutations, such as K601E, is unclear (2). These non-V600E mutations are often associated with distinct clinical behaviors and responses to therapy in NSCLC, necessitating further exploration of effective treatment strategies.

## 2 Case report

**Figure 1:**
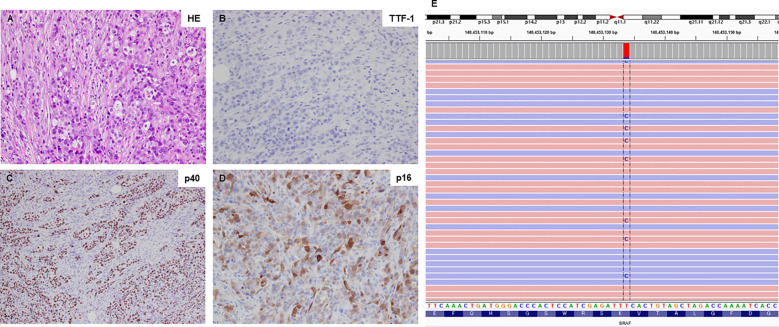
Histological findings and *BRAF* K601E sequence data. **(A)** Hematoxylin-eosin staining of the primary tumor sample (20×). **(B-D)** The primary tumor sample was negative for TTF-1 expression but positive for p40 and p16 expression (all shown at 20×). **(E)** Next-generation sequencing data illustrating the *BRAF* K601E mutation, visualized using the Integrative Genomics Viewer (IGV).

**Figure 2:**
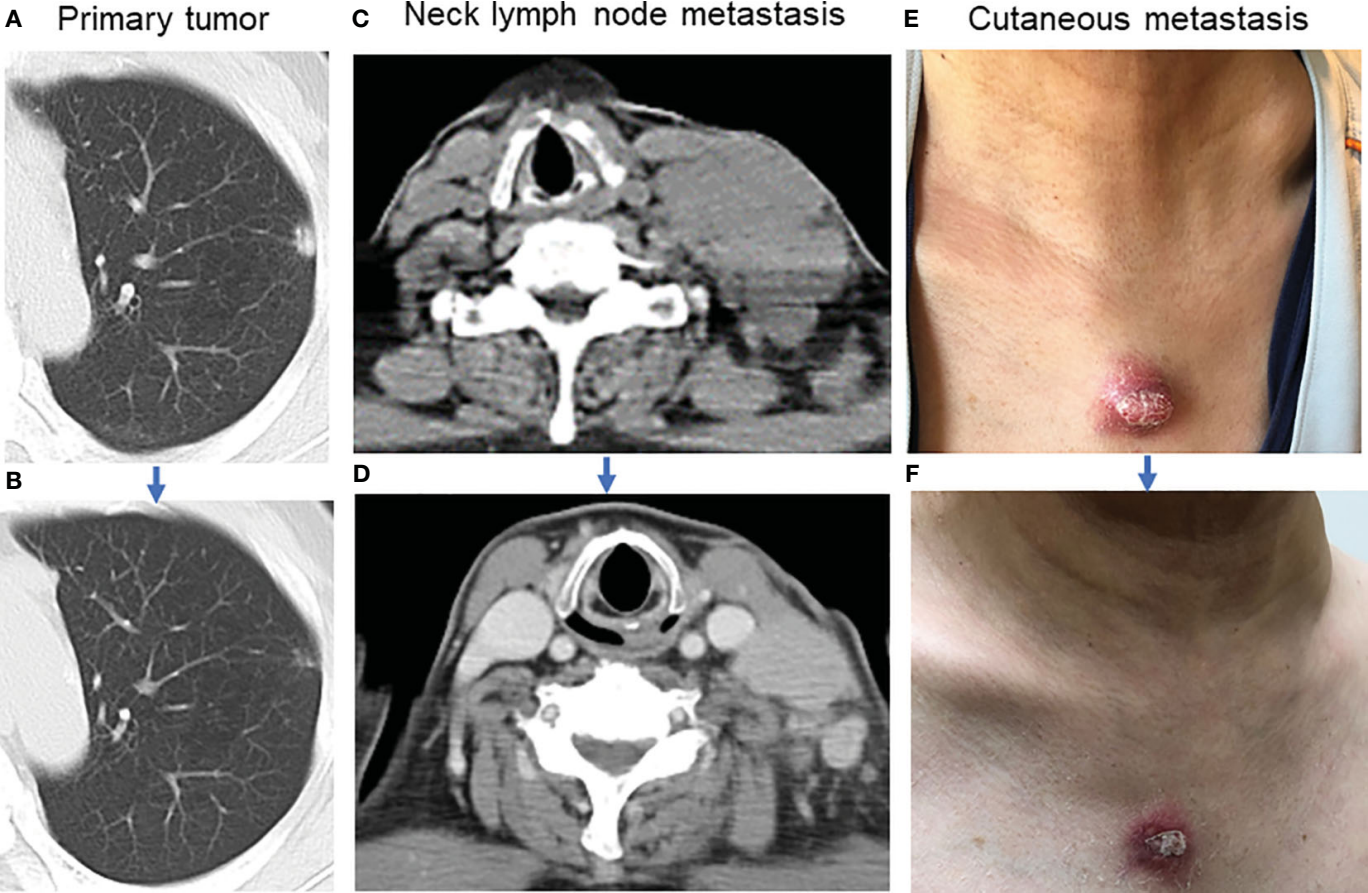
Tumor shrinkage after dabrafenib and trametinib. **(A)** Image of the primary tumor located in the left upper lobe of the lung before combination therapy. **(B)** The primary tumor almost disappeared one month after combination therapy. **(C)** Lymph node metastasis in the left neck before combination therapy. **(D)** Left neck lymph node metastasis decreased one month after combination therapy. **(E)** The appearance of the precordium cutaneous metastasis before combination therapy. **(F)** Precordium cutaneous metastasis shrank one month after combination therapy.

A 59-year-old man visited Kanazawa University Hospital with swelling in the left neck and chest stiffness. Imaging studies, including PET-CT, revealed a nodule in the upper lobe of the left lung and multiple lesions in the lymph nodes, skin, and bone. Biopsy of the skin lesions of the precordium revealed poorly differentiated carcinoma (**Figure 1A**), with negative immunostaining for TTF-1 and positive immunostaining for p40 and p16 (**Figures 1B-D**). Despite p16 positivity suggesting human papillomavirus-associated oropharyngeal cancer, no oropharyngeal findings were observed. The patient was diagnosed with poorly differentiated lung carcinoma, with programmed cell death ligand (PD-L1) expression levels ranging from 50% to 74%. Before genomic testing, the patient received carboplatin, nab-paclitaxel, and pembrolizumab as the first-line treatment. However, due to worsening pain from the growth of cutaneous metastases and deterioration of performance status, this treatment was discontinued after two courses (**Supplementary Figure 1A, B**). Genomic testing (Oncomine Dx Target Test Multi-CDx system^®^) of precordium skin metastasis (70% tumor cell content) revealed a mutation of *BRAF* K601E with a variant allele frequency of 17.4% (**Figure 1E**). Subsequently, dabrafenib (300 mg/day) and trametinib (2 mg/day) were administered as the second-line treatments. One month later, significant tumor reduction was observed (**Figure 2**), but by 1.5 months, several cutaneous metastases, including the precordium lesion, had enlarged, indicating limited treatment efficacy (**Supplementary Figures 1C**, **2**). The patient then received docetaxel and ramucirumab as third-line treatments, which were discontinued in 8 days because of severe side effects, such as disorientation or gastrointestinal bleeding.

Given the aggressive nature of his disease, he agreed to a rechallenge with dabrafenib and trametinib without a washout period. One month after the rechallenge, a liquid biopsy (Guardant360^®^) detected *BRAF* K601E, *BRAF* amplification, *KIT* amplification, and *TP53* S241F mutations (**Table 1**). However, the treatment was ineffective, and he passed away 1.5 months after the initiation of the re-challenge.

**Table 1:** Detected somatic alterations and immunotherapy biomarkers.

Detected Alterations/Biomarkers	% cfDNA or Amplification	Additional Details
*BRAF* ^K601E^	5.5%	–
*BRAF* Amplification	Medium (++) Plasma Copy Number: 2.3	–
*KIT* Amplification	Medium (++) Plasma Copy Number: 2.2	–
*TP53* ^S241F^	5.4%	–
MSI-High		Not detected

Medium (++): Amplification magnitude below the 50^th^ percentile of amplification detected by Guardant360.

MSI, microsatellite instability.

cfDNA, cell free DNA.

## 3 Discussion

This case report underscores the complexities and challenges of treating poorly differentiated lung carcinoma with the *BRAF* K601E mutation, highlighting the nuanced nature of cancer therapies, particularly in cases involving less common mutations. The initial rapid tumor regression observed with dabrafenib and trametinib treatment, followed by the development of resistance within two months, illustrates the dynamic interplay of molecular mechanisms in cancer treatment.

The resistance mechanism in our patient can be partly attributed to the nature of RAF inhibitors. These inhibitors are classified as αC-helix-IN (CI) or αC-helix-OUT (CO). CO inhibitors, such as vemurafenib and dabrafenib, are known to effectively target the monomeric form of *BRAF* V600E. However, their efficacy is significantly reduced against non-V600E mutations, such as K601E, which tend to form dimers. This dimerization can hinder the binding of CO inhibitors to the second protomer, leading to reduced efficacy (3, 4). These molecular dynamics could explain the rapid development of resistance in our patient, where the initial inhibition of monomeric BRAF K601E by dabrafenib was overcome by the emergence of dimeric forms. Interestingly, some patients with a higher variant allele frequency of *BRAF* K601E than ours exhibited a longer response to dabrafenib and trametinib (5, 6).

Additional genetic alterations identified in post-treatment liquid biopsy—*BRAF* amplification, *KIT* amplification, and the *TP53* S241F mutation—further complicate the response to treatment. Coexisting genetic changes can interact with the primary *BRAF* mutations to promote resistance. For example, *BRAF* amplification can increase the expression of mutant proteins, thereby reducing efficacy (7). Similarly, *KIT* amplification (8) and the *TP53* S241F mutation (9, 10) can activate additional oncogenic pathways or alter apoptosis, contributing to the observed resistance. In our study, both *BRAF* and *KIT* showed medium levels of amplification, suggesting that these alterations may not robustly drive resistance mechanisms owing to their moderate intensity. Additionally, targeted BRAF therapy may induce alterations in the MAPK pathway, such as the emergence of *NRAS* Q61K mutations (11, 12). However, these alterations were not detected in the present case. Understanding these complex interactions is crucial for developing effective treatment strategies for such patients.

The findings from this case emphasize the need for personalized treatment strategies for NSCLC, particularly for patients with rare or atypical *BRAF* mutations. The development of newer CI inhibitors or dual-action inhibitors that can target the monomeric and dimeric forms of BRAF could offer more effective treatment solutions (3, 4). Numerous ongoing clinical trials are investigating potential treatments for *BRAF* non-V600E mutations, including *BRAF* fusions (**Supplementary Table 1**). Additionally, integrating genomic profiling into routine clinical practice can help guide treatment choices and identify potential resistance mechanisms early during treatment.

In conclusion, this case of poorly differentiated lung carcinoma with the *BRAF* K601E mutation demonstrates the challenges of treating complex cancer cases. A multifaceted approach that includes advanced molecular diagnostics, personalized therapy, and continuous monitoring is essential for improving patient outcomes. This case serves as a call for further research into the molecular underpinnings of *BRAF*-mutant lung carcinomas and highlights the need for clinical trials to explore novel therapeutic agents targeting specific molecular pathways in patients with non-V600E *BRAF* mutations.

## Data availability statement

The raw data supporting the conclusions of this article will be made available by the authors, without undue reservation.

## Ethics statement

Written informed consent was obtained from the individual(s) for the publication of any potentially identifiable images or data included in this article.

## Author contributions

AN: Writing – original draft, Writing – review & editing. SS: Writing – review & editing. HS: Writing – review & editing. HK: Writing – review & editing. KY: Writing – review & editing. KO: Writing – review & editing. SN: Writing – review & editing. SY: Writing – review & editing. KM: Writing – review & editing. HI: Writing – review & editing. ST: Writing – review & editing.

## Supplementary material

Supplementary Figure 1 Changes in scout images for confirmation of therapeutic effect. (A) Scout image acquired before the initiation of first-line treatment. (B) Scout image captured after completion of two courses of the first-line treatment. (C) Scout image acquired 1.5 months after initiation of combination therapy.

Supplementary Figure 2 Progression of cutaneous metastases during combination therapy. (A) Appearance of the precordium cutaneous metastasis one month after initiation of combination therapy. (B) Notable enlargement of the precordium cutaneous metastasis 1.5 months after initiation of combination therapy. (C) Appearance of shoulder skin metastases one month after initiation of combination therapy. (D) Significant enlargement of shoulder skin metastases 1.5 months into combination therapy.

Supplementary Table 1 Ongoing clinical trials exploring potential treatments for Non-V600E *BRAF* mutations and *BRAF* fusions.
