# Supplementary figures and images for: Challenges in the treatment of BRAF K601E-mutated lung carcinoma: a case report of rapid response and resistance to dabrafenib and trametinib
Source: Front Oncol. 2024 Jul 8;14:1374594. doi: 10.3389/fonc.2024.1374594 (PMC11260700; doi:10.3389/fonc.2024.1374594)

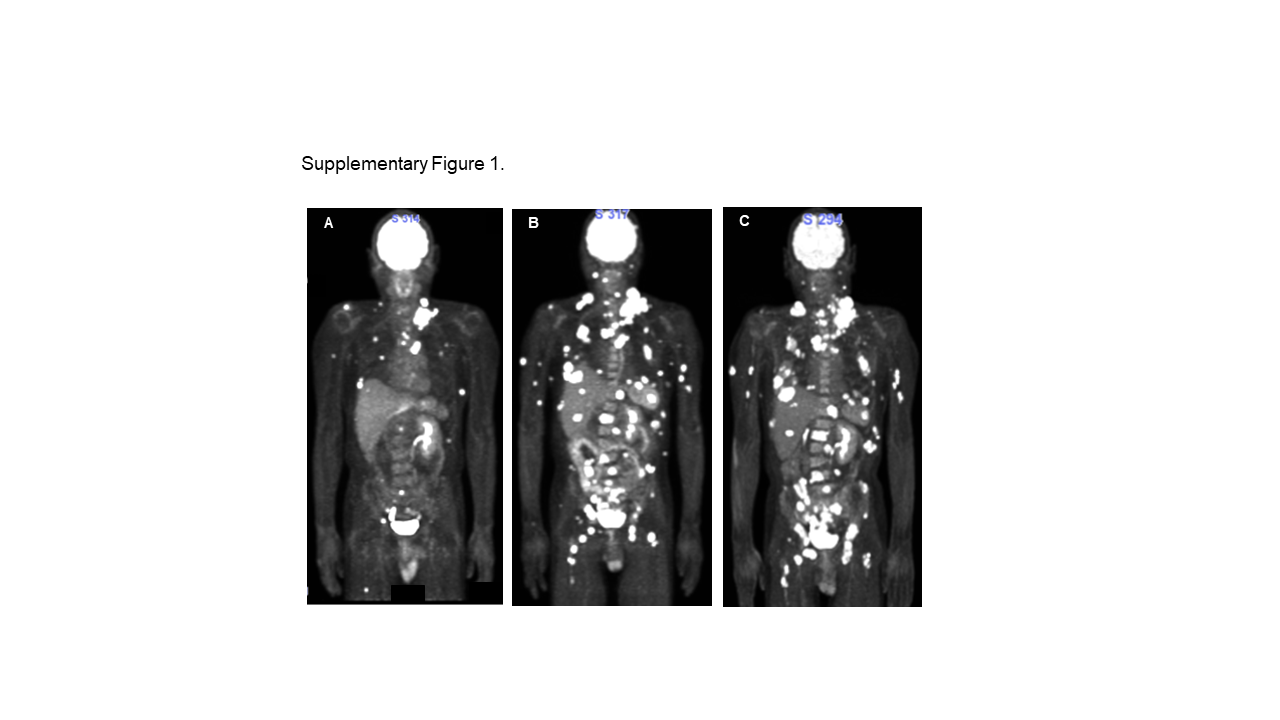

Supplement: Supplementary Figure 1 — Changes in scout images for confirmation of therapeutic effect. (A) Scout image acquired before the initiation of first-line treatment. (B) Scout image captured after completion of two courses of the first-line treatment. (C) Scout image acquired 1.5 months after initiation of combination therapy. [file Image_1.tif]

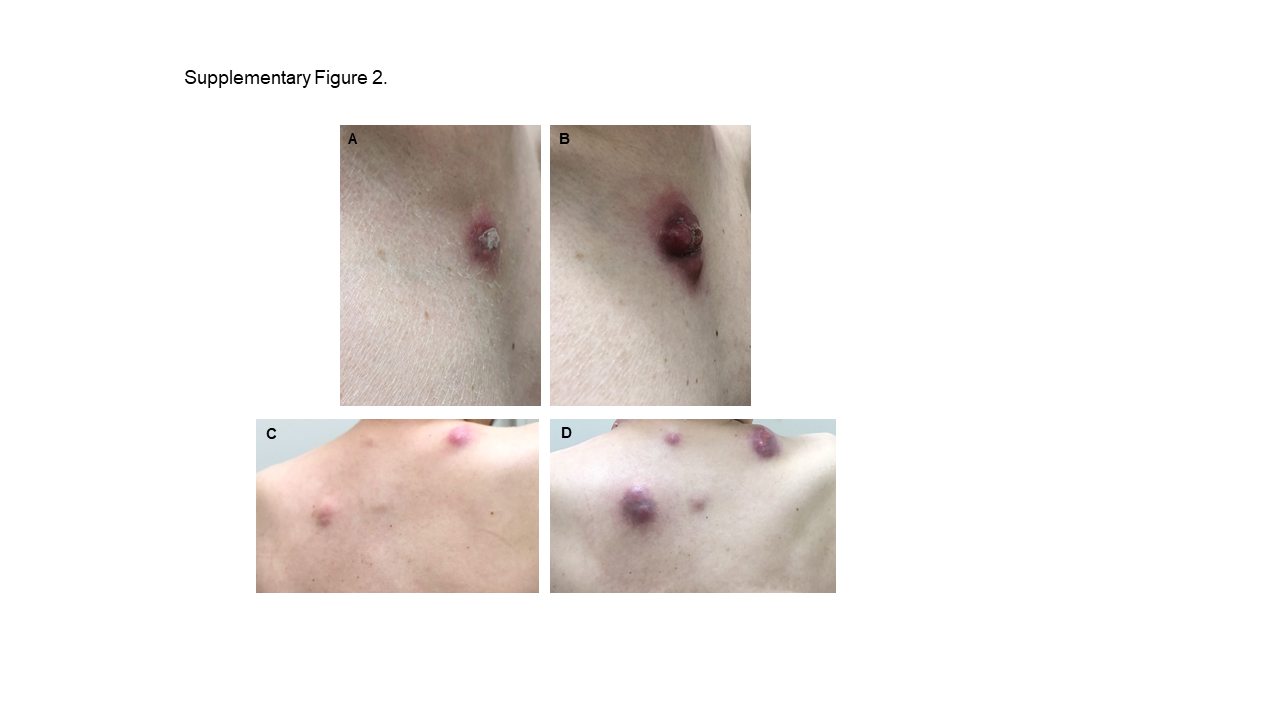

Supplement: Supplementary Figure 2 — Progression of cutaneous metastases during combination therapy. (A) Appearance of the precordium cutaneous metastasis one month after initiation of combination therapy. (B) Notable enlargement of the precordium cutaneous metastasis 1.5 months after initiation of combination therapy. (C) Appearance of shoulder skin metastases one month after initiation of combination therapy. (D) Significant enlargement of shoulder skin metastases 1.5 months into combination therapy. [file Image_2.tif]
