# Supplementary material for: Challenges in the treatment of BRAF K601E-mutated lung carcinoma: a case report of rapid response and resistance to dabrafenib and trametinib
Source: Front Oncol. 2024 Jul 8;14:1374594. doi: 10.3389/fonc.2024.1374594 (PMC11260700; doi:10.3389/fonc.2024.1374594)
Supplement: Supplementary Table 1 — Ongoing clinical trials exploring potential treatments for Non-V600E BRAF mutations and BRAF fusions. [file Table_1.docx]

**Supplementary Table 1** Ongoing clinical trials exploring potential treatments for non-V600E *BRAF* mutations and *BRAF* fusions.

| Clinical Trial | Study Design | Experimental Drug | Type of *BRAF* included | Patients population | Status |
| --- | --- | --- | --- | --- | --- |
| NCT03839342 | Open label, phase 2 single center | Encorafenib and Binimetinib | Class II and III *BRAF* | Advanced or metastatic solid tumors | Active, not recruiting |
| NCT04800822 | Open label, phase 1 | PF-07284892 | Cohort 7: Class III *BRAF* | *ALK*-positive advanced NSCLC, CRC with *BRAF* V600E mutation, *RAS* mutant, *NF-1* mutant or class III *BRAF* mutant solid tumor | Active, not recruiting |
| NCT05786924 | Open label, phase 1 | BDTX-4933 | All *BRAF* | Advanced or metastatic solid tumors or histiocytic neoplasms with *RAS* or *BRAF* mutations | Recruiting |
| NCT06208124 | Phase 1/2a | IMM-6-415 | All *BRAF* | Advanced or metastatic tumors harboring *RAS* or *RAF* oncogenic mutations | Not yet recruiting |
| NCT03284502 | Phase 1, multicenter | HM95573 with Cobimetinib or Cetuximab | *RAF* mutant solid tumors  Expansion cohort – Class II and III *BRAF* | Advanced *RAF* positive NSCLC | Active, not recruiting |
| NCT02974725 | Open label, Phase 1b, multicenter | LXH254 with LTT462 or Trametinib or Ribociclib | All *BRAF* | Advanced or metastatic NSCLC with *BRAF* or *KRAS* mutantions | Active, not recruiting |
| NCT04892017 | Open label, Phase 1/2, multicenter | DCC-3116 (monotherapy and in combination with trametinib, binimetinib, or sotorasib) | All *BRAF* | Advanced or metastatic solid tumors with RAS/MAPK pathway mutations | Recruiting |
| NCT04913285 | Open label, Phase 1/2, multicenter | KIN-2787 | All *BRAF* | Advanced or metastatic solid tumors | Recruiting |
| NCT03049618 | Phase 2a | sEphB4-HAS (fusion protein) with Pembrolizumab (anti-PD-1) | All *BRAF* | Locally advanced or metastatic NSCLC progressed after at least 1 line of platinum-based chemotherapy | Active, not recruiting |
| NCT04566393 | Expanded access | Ulixertinib (BVD-523) (ERK1/2 inhibitor) | All *BRAF* | Advanced NSCLC in altered MAPK pathway | Available |
| NCT04439279 | Phase 2 | Trametinib (MEK1/2 inhibitor) | *BRAF* fusion, *BRAF* Non-V600 | Patients with *BRAF* fusions, or non-V600E or non-V600K *BRAF* mutations | Active, not recruiting |
| NCT04249843 | Phase 1a/1b | BGB 3245 (RAF Dimer inhibitor) | Class II and Class III *BRAF* | Advanced or refractory tumor | Recruiting |
| NCT04488003 | Phase 2, multicenter | Ulixertinib (BVD-523) | *BRAF* Non-V600 | Advanced malignant tumors harboring MEK or atypical BRAF alterations | Terminated |
| NCT02428712 | Phase 1/2a | FORE8394 | *BRAF* V600 or *BRAF* Non-V600 | Advanced unresectable solid tumor | Active, not recruiting |
| NCT03843775 | Phase 1/2 | Encorafenib and Binimetinib | *BRAF* Non-V600 | Metastatic or advanced-malignant tumors | Completed |
